# *IL2RA* is associated with persistence of rheumatoid arthritis

**DOI:** 10.1186/s13075-015-0739-6

**Published:** 2015-09-08

**Authors:** H.W. van Steenbergen, J.A.B. van Nies, A. Ruyssen-Witrand, T.W.J. Huizinga, Al. Cantagrel, F. Berenbaum, A.H.M. van der Helm-van Mil

**Affiliations:** Department of Rheumatology, Leiden University Medical Center, P.O. Box 9600, 2300 RC Leiden, The Netherlands; Department of Rheumatology, Toulouse University Hospital, Toulouse, 31059 Toulouse cedex 9 France; University of Paris 06 UPMC, UMR_S-938, 75005 Paris France, Department of Rheumatology, AP-HP, Saint-Antoine Hospital, 184, rue du Faubourg-Saint-Antoine, 75012 Paris, France

## Abstract

**Introduction:**

Although rheumatoid arthritis (RA) is generally a chronic disease, a proportion of RA-patients achieve disease-modifying antirheumatic drug (DMARD)-free sustained remission, reflecting loss of disease-persistence. To explore mechanisms underlying RA-persistence, we performed a candidate gene study. We hypothesized that variants associating with lack of radiographic progression also associate with DMARD-free sustained remission.

**Methods:**

645 Dutch RA-patients were studied on DMARD-free sustained remission during a maximal follow-up duration of 10-years. Variants associated with radiographic progression under an additive model in the total RA-population (*Human Leukocyte Antigens* (*HLA)-DRB1-*shared epitope (SE), *Dickkopf-1 (DKK1)*-rs1896368, *DKK1*-rs1896367, *DKK1*-rs1528873, *C5Orf30*-rs26232, *Interleukin-2 receptor-α (IL2RA)*-rs2104286, *Matrix metalloproteinase-9* (*MMP-*9)-rs11908352, rs451066 and *Osteoprotegerin (OPG)*-rs1485305) were studied. Cox-regression analyses were performed and Bonferroni correction applied. Soluble IL2Rα (sIL2Rα)-levels were studied. For replication, 622 RA-patients included in the French Evaluation et Suivi de POlyarthrites Indifférenciées Récentes cohort (ESPOIR)-cohort were investigated. Results were combined in inverse-variance weighted meta-analysis.

**Results:**

Similar as previously reported, the SE-alleles associated with less remission (hazard ratio (HR) = 0.57, 95 % confidence interval (95 % CI) = 0.42-0.77, p = 2.72×10^−4^). Variants in *DKK-1*, *C5orf30*, *MMP-9* and *OPG* were not associated with remission. The *IL2RA*-rs2104286 minor allele associated with a higher chance on remission (HR = 1.52, 95 % CI = 1.16-1.99, p = 2.44×10^−3^). The rs2104286 minor allele associated with lower sIL2Rα-levels (p = 1.44×10^−3^); lower sIL2Rα-levels associated with a higher chance on remission (HR per 100 pg/L = 0.81, 95 % CI = 0.68-0.95, p = 0.012). When including rs2104286 and sIL2Rα-levels in one analysis, the HR for rs2104286 was 2.27 (95 % CI = 1.06-4.84, p = 0.034) and for sIL2Rα 0.83 (95 % CI = 0.70-0.98, p = 0.026). Within ESPOIR, the HR of rs2104286 was 1.31 (95 % CI = 0.90-1.90). The meta-analysis revealed a p-value of 1.01×10^−3^.

**Conclusion:**

*IL2RA*-rs2104286 and sIL2Rα-level associated with RA-persistence. *IL2RA* variants are known to protect against multiple sclerosis, diabetes mellitus and RA. Besides *HLA*-SE, *IL2RA*-rs2104286 is thus far the only known genetic variant associated with both joint destruction and RA-persistence. This underlines the relevance of *IL2RA* for RA.

**Electronic supplementary material:**

The online version of this article (doi:10.1186/s13075-015-0739-6) contains supplementary material, which is available to authorized users.

## Introduction

Persistent inflammation and progression of joint damage are the two hallmarks of rheumatoid arthritis (RA). At present, clinically relevant joint destruction has become infrequent owing to modern treatment strategies. Despite this improvement, RA is still a chronic disease in the majority of patients. Some patients, however, are able to stop taking disease-modifying antirheumatic drugs (DMARDs) without restart of DMARD treatment and without recurrence of arthritis; this is called DMARD-free sustained remission. This disease remission reflects the opposite of RA persistence and frequencies of DMARD-free sustained remission are reported to vary between 5 and 22 % [1–5]. A thorough comprehension of the mechanisms promoting disease persistence is required to derive targeted interventions aiming to reduce the chronic nature of RA. At present, however, the biologic mechanisms underlying disease persistence are largely unknown.

Only a few risk factors for RA persistence (absence of achieving DMARD-free sustained remission) have been reported and replicated. One of these factors is prolonged symptom duration at treatment start [1, 4, 6, 7]. This risk factor points to a so-called “window of opportunity” in RA but the processes underlying this association are unidentified. Another risk factor is the presence of RA-related autoantibodies [1, 2]. Although it is not exactly known how these autoantibodies exert their effect, several possibilities have been proposed [8]. However, the presence of rheumatoid factor (RF) or anti-citrullinated peptide antibodies (ACPA) explain only a proportion of the variance in achieving DMARD-free remission as the large majority of auto-antibody negative RA-patients have persistent disease and some patients with auto-antibodies can achieve remission [9]. One genetic risk factor has been found associated with arthritis persistence in two European cohorts: the presence of human leukocyte antigen (*HLA*)-*DRB1* shared epitope (SE) alleles. This risk factor presumably acts in the same pathway as ACPA [1, 2].

To increase the understanding of processes underlying disease persistence, it is valuable to study patients who have achieved DMARD-free sustained remission over time, because this reflects loss of disease persistence. This study aimed to identify further risk factors for achieving DMARD-free sustained remission. To this end, a candidate gene study was performed. To select genetic candidates, we hypothesized that genetic variants which are associated with a lack of radiographic joint damage also associate with DMARD-free sustained remission. Nine variants reported to associate with radiographic progression using an additive model in the total RA population were studied in relation to DMARD-free sustained remission in an observational cohort of 645 Dutch RA patients with a maximal follow-up of 10 years. Significant associations were evaluated for replication in a second cohort, comprising 622 French RA patients. One of the nine studied variants was the already known risk factor *HLA-DRB1* SE [1]; this variant was included in the present study for a complete overview. Another interesting gene is interleukin-2 receptor alpha (*IL2RA*); variants in *IL2RA* have shown to be associated with a decreased risk for development of RA [10, 11] and for the development of other autoimmune diseases such as multiple sclerosis (MS) [12] and diabetes mellitus (DM) [13, 14]. Furthermore, rs2104286 in *IL2RA* is, apart from the *HLA* SE, the only genetic factor that associates with the risk of RA development [10] and with the severity of radiographic progression within RA [15].

## Methods

### Patients

RA patients fulfilling the 1987 American College of Rheumatology (ACR) criteria for RA and included in two European cohorts were studied. All patients gave their informed consent, and approval was obtained from the local medical ethics committees (Medical Ethical Committee, Leiden University Medical Center and Institutional Review Board, Montpellier University Hospital).

#### Leiden Early Arthritis Clinic cohort

A total of 645 RA patients who were included between 1993 and 2008 were studied. The Leiden Early Arthritis Clinic (EAC) is a Dutch population-based inception cohort that started in 1993 and has been described previously [2]. Consecutively referred patients were included when arthritis was present at physical examination and symptom duration was <2 years. The initial treatment strategy was different for patients included and diagnosed during different inclusion periods: patients included in 1993–1995 were initially treated with nonsteroidal anti-inflammatory drugs (NSAIDs) and then DMARDs were initiated with delay; patients included in 1996–1998 were treated early with rather mild DMARDs such as hydroxychloroquine or sulfasalazine; and patients included in 1999–2008 were treated promptly with methotrexate [2].

#### Evaluation et Suivi de POlyarthrites Indifférenciées Récentes cohort

Evaluation et Suivi de POlyarthrites Indifférenciées Récentes (ESPOIR) is a prospective cohort study that started in 2002, including patients with RA or a suspicion to develop RA from 14 French rheumatology centers. Patient can be included if aged 18–70 years and at least two swollen joints are present for >6 weeks and <6 months [16]. In total, 622 RA patients consecutively included between 2002 and 2005 were studied.

In both cohorts at baseline and at the yearly follow-up visits, questionnaires were completed, physical examination was performed, and serum samples and radiographs were taken [2, 16].

### Outcome

DMARD-free sustained remission was defined as the sustained absence of arthritis (by physical examination) after discontinuation of DMARD therapy, including biologics and glucocorticoids (systemic and intra-articular). In the Leiden EAC cohort, arthritis had to be absent for the entire follow-up period and at least during 1 year. For patients with a follow-up longer than 10 years, the follow-up duration studied was restricted to 10 years. Medical files of all patients were studied on remission, and this was determined until 5 April 2012. Patients who achieved DMARD-free sustained remission initially but relapsed later over time (*n* = 2) did not fulfill the criterion that arthritis should remain absent during the total follow-up period and were included in the nonremission group. In the ESPOIR cohort, the follow-up was shorter and restricted to 5 years. To be classified as having DMARD-free sustained remission, arthritis had to be absent during at least 1 year after cessation of DMARDs but not necessarily during the rest of the follow-up. Here the outcome was assessed reviewing the structured visits in the database; medical files were not explored.

### Single nucleotide polymorphism selection and genotyping

Single Nucleotide Polymorphisms (SNPs) selection for the present study was based on a recently performed literature review on genetic variants in relation to radiographic progression [17] and the following criteria were used: (1) the SNP has been reported and replicated or found significant in meta-analysis of several cohorts to associate with radiographic joint damage progression. Furthermore, the observed association with radiographic progression was done (2) using an additive model and (3) in the total RA-population and not confined to either the ACPA-positive or ACPA-negative subgroup. The latter two criteria were included because it was expected that performing analyses on DMARD-free sustained remission using a recessive model (in which the group of patients with two minor alleles is in general small) or in only a subgroup of patients with or without autoantibodies would have insufficient power to reach statistical significance. This expectation was substantiated by power analyses (calculated using PASS 11; NCSS, Kaysville, UT, USA) based on our cohort (645 RA patients and 332 ACPA-positive patients). These analyses revealed that for an 80 % power study for an additive association in the total RA population, a hazard ratio (HR) of 1.5 would be required, but for an 80 % power study for a recessive association in the total RA population or for an additive association in only the ACPA-positive subgroup, HRs would be required of respectively 2.5 and 3.1 which are too high to be expected of SNP effects because SNPs generally have low effect sizes [11].

Based on the criteria, nine genetic variants were selected for evaluation in the Leiden EAC cohort: SE in *HLA-DRB1* [18], rs1896368, rs1896367, and rs1528873 in *Dickkopf-1* (*DKK-1*) [19], rs2104286 in *IL2RA* [15], rs26232 in *C5Orf30* [20], rs11908352 in matrix metalloproteinase-9 (*MMP-*9) [21], rs451066 at chromosome 14 [21], and rs1485305 in osteoprotegerin (*OPG*) [22]. Newly identified SNPs that were significantly associated with DMARD-free sustained remission in the Leiden EAC cohort were selected for evaluation in the ESPOIR cohort.

Within the Leiden EAC cohort, the *HLA-DRB1* alleles were genotyped using two-digit typing which was complemented by four-digit typing of the DRB1*04 alleles and by specific probes to detect the presence of the SE sequences in individuals carrying DRB1*01 or DRB1*10 alleles [1]. The following alleles were classified as SE alleles: DRB1*0101, DRB1*0102, DRB1*0104, DRB1*0401, DRB1*0404, DRB1*0405, DRB1*0408, DRB1*0413, DRB1*0416, DRB1*1001, and DRB1*1402 [23]. Genotyping data on rs1896368, rs1896367, and rs1528873 in *DKK-1* and on rs1485305 in *OPG* were retrieved using Illumina’s Golden Gate platform with an overall error rate <2.5 % and success rates >95 % [19, 22]. rs26232 in *C5Orf30* was genotyped using LightSnp (Roche) with an overall error rate <1 % and success rates >99 % [20]. Genotyping data on rs2104286 in *IL2RA*, rs11908352 in *MMP-9*, and rs451066 at chromosome 14 were retrieved using Illumina’s Immunochip with an overall error rate <1 % and success rates >98 %. Hardy–Weinberg equilibrium for all SNPs was *p* >0.001 [15, 21].

Within the ESPOIR cohort, rs2104286 was genotyped used allele-specific kinetic polymerase chain reaction analysis by KBiosciences (UK) using the KASPar method. The success rate was 97.9 % as described previously [24].

### Soluble IL2Rα

In 159 Dutch RA patients, soluble interleukin-2 receptor alpha (sIL2Rα) levels were evaluated using the standard sandwich enzyme-linked immunosorbent assay (ELISA) for sIL2Rα. The ELISA was performed according to the manufacturer’s recommendations (BD Biosciences). The serum levels were determined for a previous study on *IL2RA* [15]. Samples were collected at a median disease duration of 4 years (range 1–9 years). For patients who achieved remission, the sIL2Rα level was determined in samples taken before remission was achieved.

### Statistical analysis

Cox proportional hazard regression analyses were carried out with DMARD-free sustained remission as the outcome. The date of remission was defined as 1 year after the date at which DMARDs were withdrawn owing to remission of disease. Time to remission was the time from date of inclusion to the date of remission. Patients who did not achieve remission were censored at the date when all medical files were studied on the achievement of DMARD-free sustained remission (5 April 2012). Analyses were adjusted for age, gender, and inclusion period (a proxy for the differences over time in applied treatment strategies), similar to previous reports [2, 3, 19]. Genotypes were tested additively. The association of sIL2Rα levels (continuous variable) was tested similarly with an additional adjustment for disease duration at the time of sample collection. For the genetic variants, the cutoff for statistical significance was set at *p* <5.56 × 10^−3^ (0.05/9 tests) using the Bonferroni correction for multiple testing. For the test on the serum level, *p* <0.05 was considered significant. In the ESPOIR cohort, a similar Cox proportional hazard regression analysis adjusted for age and gender was performed. ESPOIR RA patients were diagnosed in a relatively short interval and no adjustments were made for initial treatment strategies. Results of the two cohorts were combined in an inverse variance weighted meta-analysis. Analyses were performed using IBM SPSS version 20 (Armonk, NY, USA) and STATA version 12 (College Station, TX, USA).

## Results

### Patients

The baseline characteristics of the 645 studied RA patients in the Leiden EAC cohort are presented in Table 1. During the median follow-up duration of 8.6 years (interquartile range (IQR) 5.5–10.0 years), 119 patients achieved DMARD-free sustained remission. The incidence rate for achieving remission was 2.4 per 100 person-years (119 events during the total follow-up of all patients of 4885 years). The patients who achieved remission did so after a median disease duration of 4.3 years (IQR 2.9–6.1 years). Patients who achieved DMARD-free sustained remission had shorter symptom duration at disease onset (median 12.9 versus 20.3 weeks, *p* <0.001) and had less frequent autoantibodies (ACPA-positivity, 13.0 % versus 61.3 %, *p* <0.001; RF-positivity, 27.1 % versus 64.7 %, *p* <0.001) compared with patients who did not achieve remission (Table 1).

**Table 1:** Patient characteristics of the Leiden EAC cohort

	Total (*n* = 645)	DMARD-free sustained remission achieved during follow-up (*n* = 119)	DMARD-free sustained remission not achieved during follow-up (*n* = 526)
Baseline			
Age (years), mean (SD)	56.9 (15.6)	58.8 (16.9)	56.5 (15.3)
Female, *n* (%)	430 (66.7)	74 (62.2)	356 (67.7)
Symptom duration (weeks), median (IQR)	18.8 (10.3–37.3)	12.9 (7.3–28.6)	20.3 (11.4–40.0)
Swollen joint count in 66 joints, median (IQR)	8 (4–13)	9 (4–15)	8 (4–13)
CRP level (mg/l), median (IQR)	18 (8–42)	18 (8–43)	18 (7–37)
ACPA-positive*, n* (%)	332 (52.5)	15 (13.0)	317 (61.3)
RF-positive*, n* (%)	371 (57.8)	32 (27.1)	339 (64.7)
Follow-up			
Duration until DMARD-free sustained remission (years), median (IQR)		4.3 (2.9–6.1)	N/A

*ACPA* anti-citrullinated peptide antibodies, *CRP* C-reactive protein, *DMARD* disease-modifying antirheumatic drug, *EAC* Early Arthritis Clinic, *IQR* interquartile range, *N/A* not applicable, *RF* rheumatoid factor, *SD* standard deviation

Data were missing on swollen joint count in seven patients, on CRP level in 29 patients, on ACPA in 13 patients, on RF in three patients, and on symptom duration in 47 patients

The median symptom duration and the frequencies of ACPA-positivity and RF-positivity were significantly different between patients who achieved and did not achieve DMARD-free sustained remission during follow-up (all *p* <0.001). The other baseline characteristics did not differ between the groups

### Genetic variants and achieving DMARD-free sustained remission

Presence of the SE alleles was significantly associated with DMARD-free sustained remission (*p* = 2.72 × 10^−4^). The HR per SE allele on achieving DMARD-free sustained remission was 0.57 (95 % confidence interval (95 % CI) = 0.42–0.77) compared with patients without SE alleles (Table 2; Additional file 1); this finding is in line with previous reports.

**Table 2:** Genetic risk factors for severity of joint damage in relation to achieving DMARD-free sustained remission in the Leiden EAC cohort

Genetic variant (minor allele)	Located in/nearby gene(s) (chromosomes)	MAF (%)	HR per minor allele (95 % CI)	*p* value
Shared epitope [18]	*HLA-DRB1* (6)	39.6	0.57 (0.42–0.77)	2.72 × 10^−4^
rs1896368 (G) [19]	*DKK-1* (10)	45.8	0.98 (0.75–1.28)	0.88
rs1896367 (A) [19]		41.5	0.96 (0.73–1.25)	0.75
rs1528873 (C) [19]		46.7	1.21 (0.93–1.58)	0.15
rs2104286 (C) [15]	*IL2RA* (10)	24.3	1.52 (1.16–1.99)	2.44 × 10^−3^
rs26232 (T) [20]	*C5orf30* (5)	28.9	1.08 (0.81–1.44)	0.61
rs11908352 (A) [21]	*MMP-9* (20)	20.9	0.78 (0.56–1.09)	0.15
rs451066 (A) [21]	rs1465788 (14)	19.6	0.87 (0.63–1.21)	0.41
rs1485305 (T) [22]	*OPG* (8)	44.2	1.00 (0.76–1.32)	0.98

*CI* confidence interval, *DKK-1* Dickkopf-1, *HLA* human leukocyte antigen, *HR* hazard ratio, *IL2RA* interleukin-2 receptor alpha, *MAF* minor allele frequency, *MMP-9* matrix metalloproteinase-9, *OPG* osteoprotegerin

Analyses were adjusted for age, gender, and inclusion period (as proxy for treatment strategy)

rs1896368, rs1896367, and rs1528873 (all *DKK-1*), rs26232 (*C5Orf30*), rs11908352 (*MMP-9*), rs451066 (chromosome 14), and rs1485305 (*OPG*) were not associated with DMARD-free sustained remission (Table 2).

**Fig. 1:**
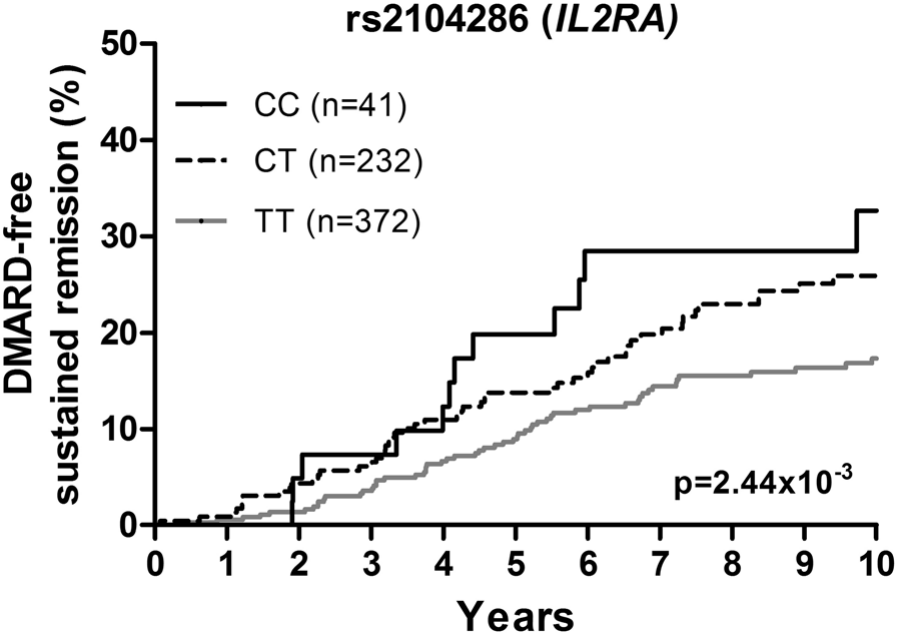
rs2104286 in *IL2RA* in relation to achieving DMARD-free sustained remission in RA patients of the Leiden EAC cohort. rs2104286 in *IL2RA* was significantly associated with achieving DMARD-free sustained remission in 645 RA patients (*p* = 2.44 × 10^−3^). The HR per minor C allele for achieving remission was 1.52 (95 % CI = 1.16–1.99). The analysis was adjusted for age, gender, and inclusion period (as a proxy for treatment strategy). *DMARD* disease-modifying antirheumatic drug, *IL2RA* interleukin-2 receptor alpha

rs2104286 in *IL2RA* significantly associated with achieving DMARD-free sustained remission (*p* = 2.44 × 10^−3^). The HR per minor C allele for achieving DMARD-free sustained remission was 1.52 (95 % CI = 1.16–1.99) compared with the reference genotype of patients who were homozygous for the major T allele (Table 2 and Fig. 1); hence patients with the minor allele had an increased chance of achieving remission and, as reported earlier [15], less radiographic progression.

### Genetic variants and achieving DMARD-free sustained remission in relation to ACPA status

Because genetic risk factors for ACPA-positive and ACPA-negative RA are different and ACPA-positive and ACPA-negative RA are considered separate disease entities, we studied whether the observed associations were independent of ACPA or were restricted to a subset of RA patients. The analyses of *HLA-DRB1* SE and rs2104286 (*IL2RA*) were therefore repeated with additional adjustment for ACPA and when stratifying for ACPA status.

When including both SE and ACPA in one analysis, SE was not significantly associated (HR = 0.92, 95 % CI = 0.67–1.26, *p* = 0.61) whilst ACPA remained significant (HR = 0.13, 95 % CI = 0.072–0.22, *p* = 7.68 × 10^−13^), suggesting that ACPA act in the path of the SE alleles and DMARD-free sustained remission. Similarly, the SE alleles were not associated with remission in the ACPA-positive and ACPA-negative subgroups separately (*p* = 0.84 and *p* = 0.51 respectively; Figure S2A in Additional file 2).

Adding ACPA as additional adjustment factor in the analysis of rs2104286 (*IL2RA*) in relation to DMARD-free sustained remission revealed an HR for rs2104286 of 1.47 (95 % CI = 1.12–1.93, *p* = 5.78 × 10^−3^), suggesting that the association of rs2104286 with remission is independent of ACPA. Stratified analysis on rs2104286 in ACPA-positive and ACPA-negative subgroups showed an HR of 1.82 (95 % CI = 0.88–3.77, *p* = 0.11) within ACPA-positive RA and an HR of 1.41 (95 % CI = 1.05–1.89, *p* = 0.024) within ACPA-negative RA (Figure S2B in Additional file 2).

### sIL2Rα levels and achieving DMARD-free sustained remission

Previous studies have shown correlations between rs2104286 in *IL2RA* and IL2Rα serum levels [25, 26]. Similarly, we have previously studied rs2104286 in *IL2RA* and sIL2Rα levels in 159 RA patients from the Leiden EAC cohort and observed a significant association; the rs2104286 minor allele associated with lower sIL2Rα levels (*p* = 1.44 × 10^−3^) [15]. We then explored whether sIL2Rα levels were also associated with DMARD-free sustained remission and observed that lower serum levels were indeed associated with more remission (*p* = 0.012); per 100 pg/ml increase in level, the HR of achieving remission was 0.81 (95 % CI = 0.68–0.95). In the 159 patients with information on sIL2Rα, rs2104286 was also associated with DMARD-free sustained remission (HR = 2.57, 95 % CI = 1.20–5.50, *p* = 0.015). An analysis including both rs2104286 and sIL2Rα revealed an HR of 2.27 for rs2104286 (95 % CI = 1.06–4.84, *p* = 0.034) and a HR (per 100 pg/ml) of 0.83 for sIL2Rα (95 % CI = 0.70–0.98, *p* = 0.026).

### Replication of rs2104286 in relation to DMARD-free sustained remission in the ESPOIR cohort

**Fig. 2:**
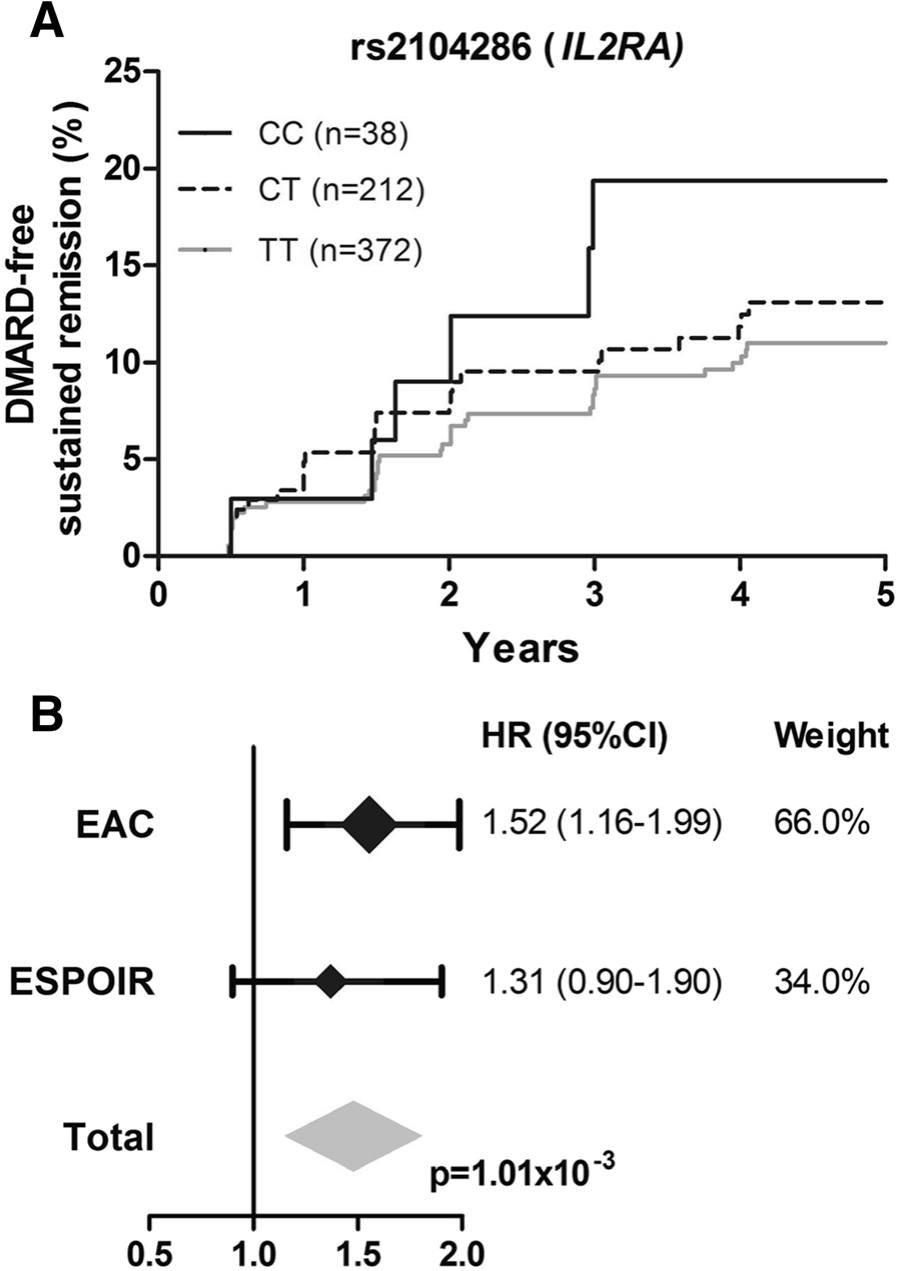
rs2104286 in *IL2RA* in relation to achieving DMARD-free sustained remission in RA patients of the ESPOIR cohort and in meta-analysis of the Leiden EAC and ESPOIR cohorts. **a** In 622 RA patients of the ESPOIR cohort, the HR per minor C allele for achieving remission was 1.31 (95 % CI = 0.90–1.90, *p* = 0.16). The analysis was adjusted for age and gender. The minor allele frequency in the ESPOIR cohort was 23.2 %. **b** Results of the Leiden EAC and ESPOIR cohorts were combined in an inverse variance weighted meta-analysis: *I* ^2^ = 0.0 %, *p* = 0.53, fixed-effect *p* = 1.01 × 10^−3^, random-effects *p* = 1.01 × 10^−3^. *CI* confidence interval, *DMARD* disease-modifying antirheumatic drug, *EAC* early arthritis clinic, *ESPOIR* Evaluation et Suivi de POlyarthrites Indifférenciées Récentes, *HR* hazard ratio, *IL2RA* interleukin-2 receptor alpha

Subsequently, rs2104286 in *IL2RA* was studied for replication in 622 French RA patients. The mean (standard deviation) age was 48.8 (12.3) years, 76 % were female, the median (IQR) symptom duration was 22 (13–33) weeks, and 46 % were ACPA-positive. After a median (IQR) follow-up duration of 5.0 (3.0–5.0) years, 67 patients achieved DMARD-free sustained remission after a median follow-up duration of 1.5 (0.7–3.0) years. The incidence rate for remission was 2.7 per 100 person-years (67 events/2451 years of total follow-up in all patients). The number of events (*n* = 67) was lower than that of the first cohort, so the power to find significance was expected to be less than that of the first phase. Evidence of a tendency in the same direction was still considered relevant and Cox regression analyses on rs2104286 and remission were performed. The HR per minor C allele for achieving DMARD-free sustained remission was 1.31 (95 % CI = 0.90–1.90, *p* = 0.16) compared with the common genotype. Although not reaching statistical significance, this indicates that, similar to the Leiden EAC cohort, patients with the minor allele had an increased chance of achieving remission (Fig. 2a). When additionally adjusting the analysis for ACPA, the HR was 1.37 (95 % CI = 0.95–1.97, *p* = 0.097). Meta-analysis of the results of the Leiden EAC and ESPOIR cohorts revealed a fixed-effect *p* value of 1.01 × 10^−3^ (Fig. 2b).

## Discussion

The biological mechanisms driving disease chronicity in RA are largely unidentified. We therefore aimed to determine genetic risk factors for disease persistence in RA. Because of the low frequency of DMARD-free sustained remission (reflecting loss of disease persistence) and because of the lack of multiple large cohorts with data on this disease outcome, we were not able to perform a hypothesis-free genome-wide association study or to analyze the whole Immunochip. We used a candidate gene approach instead and hypothesized that genetic variants which associated with the severity of joint damage also associated with disease persistence. In addition to the previously reported association between the *HLA-DRB1* SE alleles and DMARD-free sustained remission (reflecting loss of disease persistence), we demonstrated that rs2104286 in *IL2RA* associated with DMARD-free sustained remission; this minor allele that was previously associated with less severe radiographic progression [15] was associated with a higher chance of DMARD-free sustained remission. Also, the lower level of sIL2Rα observed in the presence of the rs2104286 minor allele associated with a higher chance of DMARD-fee sustained remission. Altogether the present data from two observational cohorts indicate that the *IL2RA* minor allele is not only protective for the severity of radiographic progression but also predisposes to a less persistent course of RA.

*IL2RA* encodes the α-chain of the high-affinity IL-2 receptor (CD25) which is expressed on and upregulated after stimulation in many immune cells, including regulatory T cells (Tregs) [27, 28]. Variants in *IL2RA* are also associated with the risk of development of RA [10, 11] and other autoimmune diseases such as MS [12] and type 1 DM [13, 14]. sIL2Rα is produced by proteolytic cleavage of cell-bound IL2Rα and is considered reflective of the extent of activation and expansion of T cells [26, 29, 30]. Other studies reported that the minor allele of rs2104286 correlated with lower sIL2Rα levels in patients and healthy individuals [15, 25, 26]. In our previous study, rs2104286 was no longer associated with joint destruction after including sIL2Rα in the analysis, suggesting that the SNP might act in the same path that influenced the serum levels [15]. In present study, the genetic and serological marker remained significantly associated with DMARD-free sustained remission. This might suggest that *IL2RA* exerts part of its effect by a path which does not influence sIL2Rα levels. However, association studies cannot answer causality questions.

RA is considered to consist of ACPA-positive and ACPA-negative subentities, each with different genetic risk variants [31, 32]. To determine whether the observed association was present in one or both subsets, stratified analyses were performed. Although these analyses were assumed to have insufficient power (owing to lower number of patients, and in ACPA-positive RA also a low frequency of remission), they were performed to gain insight into the data. Adjusting for ACPA is more powerful. The association of rs2104286 with DMARD-free sustained remission was independent of ACPA.

In the present study we did not fine-map the *IL2RA* region in relation to DMARD-free sustained remission because we expected to have insufficient power to find statistical significance after correcting for >400 tests. Previously the *IL2RA* region was fine-mapped in relation to joint damage progression, which is a more powerful analysis than the present survival analysis because it makes use of repeated measurements over time. rs12722508 was identified as the SNP with the strongest association [15]. Evaluating rs12722508 in relation to DMARD-free sustained remission revealed a lower *p* value and larger HR for rs12722508 (HR = 1.93, *p* = 7.90 × 10^−4^) compared with rs2104286 (HR = 1.52, *p* = 2.44 × 10^−3^). Although fine-mapping was not performed in this study, these data strengthen the finding on *IL2RA* and DMARD-free sustained remission.

At present, there is not much literature on the description of RA persistence or chronicity. In the present study, patients who were unable to reach DMARD-free sustained remission were considered to have persistent disease. Although other definitions for RA persistence can be used, we have chosen the absence of DMARD-free sustained remission as the outcome because it is a strict definition and the closest available proxy for cure of the disease.

The majority of the studied patients had persistent disease and did not achieve remission. Recently, we reported that the chance of achieving DMARD-free sustained remission in clinical practice has become a more feasible outcome with up-to-date treatment strategies [5]. The Leiden EAC patients who were evaluated in the present study were included during the period 1993–2006. Treatment strategies have changed over time in these patients and indeed patients included in later periods had a higher chance of achieving DMARD-free sustained remission (data not shown). All analyses in the present study were adjusted for the inclusion period as a proxy for the initially applied treatment strategy, and the results obtained for *IL2RA* were thus independent of the effect of changes in treatment strategies.

Another potential limitation is that we evaluated data from longitudinal observational cohort studies. These data reflect the daily care of patients and not only decisions to start DMARDs but also decisions to stop DMARDs were left to the patients’ and rheumatologists’ decisions and not protocolized. In the ESPOIR cohort, mainly in the first years of its existence, quitting DMARD therapy was uncommon. Consequently, the observed frequency of DMARD-free sustained remission may be underestimated. This may be one of the explanations contributing to a lower incidence of DMARD-free sustained remission in the ESPOIR cohort. In addition, whether DMARD-free sustained remission was achieved was determined slightly differently in the cohorts. In the Leiden EAC cohort, all medical files were checked to ensure that DMARD-free sustained remission was present. In the ESPOIR cohort, data from the structured visits with yearly intervals were studied. It is possible that more patients included in the ESPOIR cohort would have achieved DMARD-free sustained remission when all information present in medical files was evaluated. Thirdly, the follow-up duration was shorter in the ESPOIR cohort. Differences in common practice for discontinuing DMARD therapy, however, might be the most important cause for the higher frequency of DMARD-free sustained remission in the Leiden EAC cohort than in the ESPOIR cohort. Nonetheless, there was a strong tendency in the data from the ESPOIR cohort validating the importance of *IL2RA* for the disease course in RA.

The SE alleles were strongly associated with sustained DMARD-free remission. A similar result was previously reported (although using a dominant model instead of an additive model). We here observed that this association was not independent of ACPA, suggesting that the SE alleles act in the same path as ACPA. This finding is similar to that observed for SE, ACPA, and radiographic progression [33].

The studied variants in *DKK-1*, *C5Orf30*, *MMP-9*, and *OPG* were not associated with DMARD-free sustained remission. Although power issues might have contributed to some negative findings, the absence of an association of these risk factors for radiographic progression with DMARD-free sustained remission suggests that the mechanisms driving joint damage progression and disease persistence are partially different.

At present >100 genetic susceptibility factors are known and several genetic risk factors for radiographic progression have been identified [11, 34]. These factors were largely dissimilar; only the *HLA-DRB1* SE alleles and *IL2RA* were present in both lists of risk factors. Interestingly, the current study determined that both factors are also associated with persistence of RA. This suggests that both variants are of crucial importance for the processes mediating RA development and progression.

IL-2/IL-2 receptor signaling is important during immune responses of both effector T cells and Tregs. Quantitatively, Tregs require less IL-2/IL-2 receptor signaling than effector T cells to support their development and function [35, 36]. Recently, the first results on immunomodulation with low-dose IL-2 in other autoimmune diseases have been published, showing efficacy on upregulation of Tregs [34, 35] and improved clinical outcome [37]. Monoclonal anti-CD25 antibodies (daclizumab) have also been shown effective in reducing disease activity in autoimmune diseases [38]; this effect is not only ascribed to direct effects on T cells but also on natural killer (NK) cells and dendritic cells [38]. To the best of our knowledge there are no data on IL-2 treatment for RA. However, if low-dose IL-2 treatment is effective, the results of IL-2 therapy in RA might also be dependent on the IL-2 receptor status of the patient, which is genetically determined. Hence, the *IL2RA* genotype presumably affects the response of IL-2 therapy and might be relevant for personalized medicine.

## Conclusion

Genetic studies are useful because they can point to mechanisms that are pivotal for disease development or disease progression. This study observed that rs2104286 in *IL2RA* and the sIL2Rα level are associated with RA persistence. Besides the *HLA-DRB1* SE, *IL2RA* is the only genetic risk factor for development of RA and for both radiographic progression and persistence. This underlines the relevance of *IL2RA* for RA. Further research is needed to gain more insight into the underlying mechanisms of arthritis persistence.

## Additional files

Additional file 1: Figure S1. Showing SE alleles in *HLA-DRB1* in relation to achieving DMARD-free sustained remission in RA patients in the Leiden EAC cohort. SE alleles were significantly associated with achieving DMARD-free sustained remission in 616 RA patients with *HLA-DRB1* genotyping data (*p* = 2.72 × 10^−4^). The HR per SE allele for achieving remission was 0.57 (95 % CI = 0.42–0.77). The analysis was adjusted for age, gender, and inclusion period (as proxy for treatment strategy). (PDF 52 kb) Additional file 2: Figure S2. Showing genetic variants in relation to achieving DMARD-free sustained remission in ACPA-positive and ACPA-negative RA patients from the Leiden EAC cohort. SE in ACPA-positive: HR per SE allele = 0.92 (95 % CI = 0.42–2.03). SE in ACPA-negative: HR per SE allele = 0.89 (95 % CI = 0.63–1.25). rs2104286 (*IL2RA*) in ACPA-positive: HR per minor allele = 1.82 (95 % CI = 0.88–3.77). rs2104286 (*IL2RA*) in ACPA-negative: HR per minor allele = 1.41 (95 % CI = 1.05–1.89). All analyses were adjusted for age, gender, and inclusion period (as proxy for treatment strategy). Note that the *y* axes of the ACPA-positive and ACPA-negative subgroups are different. (PDF 218 kb)

## Abbreviations

ACPA: Anti-citrullinated peptide antibodies
ACR: American College of Rheumatology
CI: Confidence interval
DKK-1: Dickkopf-1
DM: Diabetes mellitus
DMARD: Disease-modifying antirheumatic drug
EAC: Early Arthritis Clinic
ELISA: Enzyme-linked immunosorbent assay
ESPOIR: Evaluation et Suivi de POlyarthrites Indifférenciées Récentes cohort
HLA: Human leukocyte antigen
HR: Hazard ratio
IL2RA: Interleukin-2 receptor alpha
IQR: Interquartile range
MMP-9: Matrix metalloproteinase-9
MS: Multiple sclerosis
NK: Natural killer
NSAID: Nonsteroidal anti-inflammatory drug
OPG: Osteoprotegerin
RA: Rheumatoid arthritis
RF: Rheumatoid factor
SE: Shared epitope
sIL2Rα: Soluble interleukin-2 receptor alpha
SNP: Single nucleotide polymorphism
Tregs: Regulatory T cells
