# Supplementary material for: IL2RA is associated with persistence of rheumatoid arthritis
Source: Arthritis Res Ther. 2015 Sep 8;17(1):244. doi: 10.1186/s13075-015-0739-6 (PMC4563834; doi:10.1186/s13075-015-0739-6)
Supplement: Additional file 1: Figure S1. — Showing SE alleles in HLA-DRB1 in relation to achieving DMARD-free sustained remission in RA patients in the Leiden EAC cohort. SE alleles were significantly associated with achieving DMARD-free sustained remission in 616 RA patients with HLA-DRB1 genotyping data (p = 2.72 × 10^−4^). The HR per SE allele for achieving remission was 0.57 (95 % CI = 0.42–0.77). The analysis was adjusted for age, gender, and inclusion period (as proxy for treatment strategy). (PDF 52 kb) [file 13075_2015_739_MOESM1_ESM.pdf]

Additional file 1. Shared epitope alleles in *HLA-DRB1* in relation to achieving DMARD-free sustained remission in rheumatoid arthritis patients in the Leiden EAC

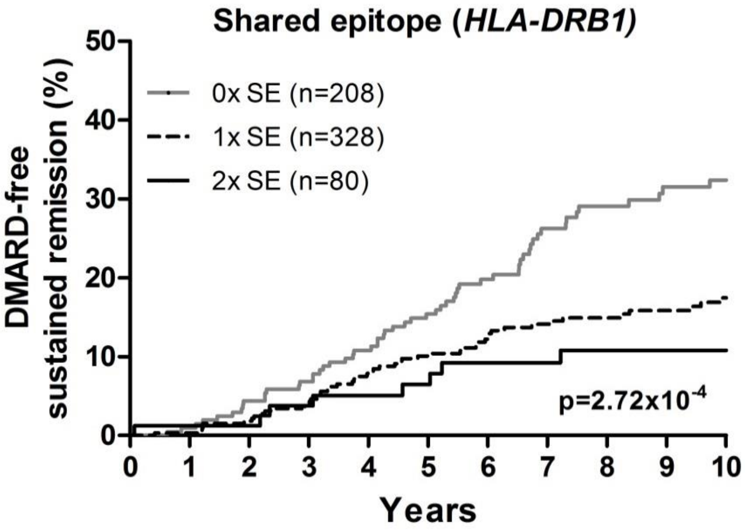

The shared epitope alleles were significantly associated with achieving DMARD-free sustained remission in 616 rheumatoid arthritis patient with *HLA-DRB1* genotyping data ($p=2.72x10^{-4}$). Per shared epitope allele the hazard ratio for achieving remission was 0.57 (95% confidence interval 0.42-0.77). The analysis was adjusted for age, gender and inclusion period (as proxy for treatment strategy).
