# Supplementary material for: IL2RA is associated with persistence of rheumatoid arthritis
Source: Arthritis Res Ther. 2015 Sep 8;17(1):244. doi: 10.1186/s13075-015-0739-6 (PMC4563834; doi:10.1186/s13075-015-0739-6)
Supplement: Additional file 2: Figure S2. — Showing genetic variants in relation to achieving DMARD-free sustained remission in ACPA-positive and ACPA-negative RA patients from the Leiden EAC cohort. SE in ACPA-positive: HR per SE allele = 0.92 (95 % CI = 0.42–2.03). SE in ACPA-negative: HR per SE allele = 0.89 (95 % CI = 0.63–1.25). rs2104286 (IL2RA) in ACPA-positive: HR per minor allele = 1.82 (95 % CI = 0.88–3.77). rs2104286 (IL2RA) in ACPA-negative: HR per minor allele = 1.41 (95 % CI = 1.05–1.89). All analyses were adjusted for age, gender, and inclusion period (as proxy for treatment strategy). Note that the y axes of the ACPA-positive and ACPA-negative subgroups are different. (PDF 218 kb) [file 13075_2015_739_MOESM2_ESM.pdf]

Additional file 2. Genetic variants in relation to achieving DMARD-free sustained remission in ACPA-positive and ACPA-negative rheumatoid arthritis patients from the Leiden EAC

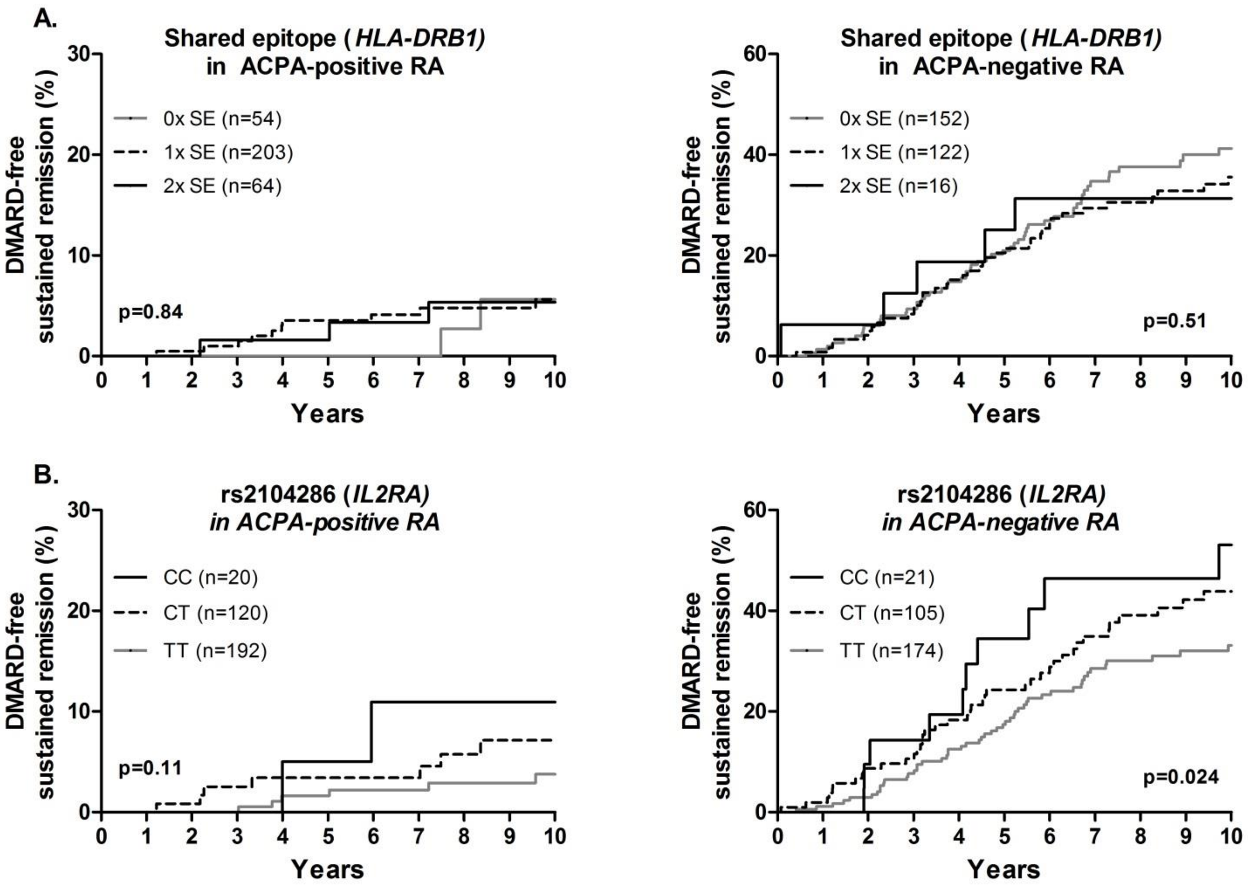

Shared epitope (SE) in ACPA-positive: hazard ratio (HR) per SE-allele is 0.92 (95% confidence interval (95%CI)=0.42-2.03). SE in ACPA-negative: HR per SE-allele is 0.89 (95%CI=0.63-1.25). Rs2104286 (*IL2RA*) in ACPA-positive: HR per minor allele is 1.82 (95%CI=0.88-3.77). Rs2104286 (*IL2RA*) in ACPA-negative: HR per minor allele is 1.41 (95%CI=1.05-1.89). All The analyses were adjusted for age, gender and inclusion period (as proxy for treatment strategy). Note that the y-axes of the ACPA-positive and ACPA-negative sub groups are different.
